# Mode of delivery alters sensitivity to thermal and chemical stimuli in adult rats: An experimental study

**DOI:** 10.18502/ijrm.v22i2.15707

**Published:** 2024-03-25

**Authors:** Parastoo Nikkhouy, Mehdi Abbasnejad, Saeed Esmaeili-Mahani, Razieh Kooshki

**Affiliations:** ^1^Department of Biology, Faculty of Sciences, Shahid Bahonar University of Kerman, Kerman, Iran.; ^2^Laboratory of Molecular Neuroscience, Kerman Neuroscience Research Center, Institute of Neuropharmacology, Kerman University of Medical Sciences, Kerman, Iran.; ^3^Department of Biology, Faculty of Sciences, Lorestan University, Khorramabad, Iran.

**Keywords:** Childbirth, C-section, Nociception, Inflammation, Rats.

## Abstract

**Background:**

The mode of delivery might prompt a long-lasting alteration in physiological and behavioral responsiveness in offspring.

**Objective:**

This study was intended to evaluate if the mode of delivery could alter sensitivity to thermal and chemical stimuli in female rats.

**Materials and Methods:**

56 adult female Wistar rats (200–220 gr) that were born by vaginal or cesarean section (C-section) were used (n = 28/each). Inflammatory pain was induced by subcutaneous injection of formalin into the hind paw. The thermal nociceptive threshold was determined by tail-flick and hot plate tests. Besides, the Western blot test was used to evaluate the spinal cord levels of c-Fos and c-Jun proteins.

**Results:**

Formalin-induced inflammation was significantly decreased in C-section group as compared to vaginally born rats (p < 0.001). The baseline nociceptive threshed and morphine-induced analgesia were significantly increased in C-section groups in comparison to vaginally born rats. In addition, the levels of c-Fos and c-Jun proteins were significantly decreased in the spinal cord of C-section rats as compared to vaginally born animals (p < 0.01). Morphine treatment could decrease the expression of c-Fos and c-Jun in the C-section group (p < 0.05).

**Conclusion:**

Overall, C-section rats showed lower spinal nociceptive processing and neuronal activity later in life, compared to the vaginal born rats.

## 1. Introduction

Cesarean section (C-section) primarily took place due to obstetrical complications or medical illness. However, in addition to inevitable cases, the tendency to elective primary C-sections with no apparent medical or obstetrical indication has intensely increased in recent years (1, 2).

The neonatal period is essential for the development of brain structures. Much-related evidence shows that the brain's physiological function is affected by changes in brain development after childbirth (3, 4). Especially, the pain threshold in newborns is lower than that in adults due to the incomplete development of the sensory pathways (5).

It has been indicated that physiological function, including pain sensitivity, is somewhat affected by the type of delivery. The risk of postpartum infection and pulmonary embolism has been higher in planned C-sections than with vaginal delivery (6).

In addition, infants born by cesarean delivery have been shown to have a deficiency of immune system function (7). Instead, it has been found that the incidence of learning disabilities in cesarean children under neuraxial anesthesia is lower than vaginal delivery (8). C-section-delivered rats have been shown to have lower sensitivity to capsaicin-evoked pulpal nociception. Moreover, learning and memory performances are improved in C-sections than vaginally delivered rats (9). The mode of delivery changes the plasma concentration of opioid peptides in both mothers and infants (10).

Peripheral noxious stimulation has been shown to increase c-Fos protein expression in rats' lumbar neurons of spinal cord. The c-Fos and c-Jun proteins complex is involved in the regulation of transcription from promoters containing nuclear factor activator protein 1 (11, 12).

This study primarily evaluated whether a type of delivery, vaginal or C-section, could alter sensitivity to thermal or chemical stimuli in female rats in later life. In addition, the delivery-related changes in neuronal activity and nociception were evaluated by assessing c-Fos and c-Jun protein levels within the lumbar spinal cord of rats. Together, the aim of the current study was to evaluate the probable differences between offspring of this 2 type of delivery methods in rats.

## 2. Materials and Methods

### Animals

In this experimental study, 20 pregnant Wistar rats were used. The study was processed. The animals were preserved in the animal room of the Department of Biology, Shahid Bahonar University, Kerman, Iran. 9 rats gave birth vaginally, and 11 underwent C-sections. After giving birth, each mother with her offspring was kept in an individual cage until the 4 ${\;}^{\text{th}}$ wk. Then, the female pups were moved in 4 per cage. A total of 76 female pups were involved. The pups were controlled for exclusion criteria, including abnormal behavioral performance and motor deficiency. None of the rats were excluded. The animals were housed in the same place under a 12-hr light/dark cycle with temperature-controlled at 22 ± 2 C. Food and water were available *ad libitum*. 56 adult female offspring (2 months, 200–220 gr) born by vaginal or cesarean procedure were used in experiments (n = 28/each). The rats were randomly divided into 8 groups (n = 7 in each group).

### Cesarean procedure

The pregnant rats were transferred to the operation lab 21 days after the beginning of pregnancy. The rats were anesthetized with an intra-parietal injection of a mixture of ketamine (Alphason-Netherlands) (65 mg/kg) and xylazine (Alphason-Netherlands) (10 mg/kg); hysterectomies were performed to remove the uterine. Then, through a lateral incision of the uterine, the pups and all of their amniotic membrane were externalized. The mother's abdomen incision was closed with a sterile needle and thread stitches. Lastly, the mothers and pups were placed in a cage to feed and remove the placenta.

### Formalin test

Formalin (2.5%) (Merck, USA) produced biphasic pain in the rat's paw. Following formalin injection, the rats were placed in a 30 × 30 × 30 cm plexiglas box with a mirror on the floor at a 45 angle. The test consisted of phase 1 (0–10 min) and phase 2 (15–60 min). In each stage, cumulative time of biting and licking behaviors were recorded as nociceptive time (13). Moreover, 2 groups of rats were injected intraperitoneally with ibuprofen (10 mg/kg) 20 min before formalin injection to evaluate anti-inflammatory response.

### Thermal nociception tests

The tail flick and hot plate tests were used to evaluate basal nociceptive threshold and morphine-induced thermal analgesia in rats. The morphine injection (10 mg/kg) was performed 20 min before nociceptive assessment.

#### Tail-flick test

The rats were gently controlled, and radiant heat was applied to the base of the tail. Tail-flick withdrawal latency time was verified for each animal 3 times with 2 min intervals between experiments, and the meantime was described. A cutoff time was set at 12 sec to avoid tissue injury (14).

#### Hot plate test

The hot plate test was used to measure thermal hyperalgesia. The rats were placed individually on a metal surface maintained at a constant temperature (52 ± 2 C). The latencies for licking, lifting, or jumping responses were recorded. And the mean of 3 times of the 1 ${\;}^{\text{st}}$ animal reaction was reported as a test response. To avoid tissue damage, the cut-off time was considered 30 sec (15).

### Western blot test 

The lumbar portion of the rat's spinal cord was homogenized at radioimmunoprecipitation assay buffer. Then, the sample was centrifuged at 12,000 × g for 20 min at 4 C, and heated for 5 min at 100 C in Laemmli sample buffer. An equal amount (40 µg) of the sample was electrophoresed in sodium dodecyl sulfate-tris-glycine polyacrylamide gels for 90 min at 100 constant volts. After that, the proteins were transferred in an electrical field from the gel onto a polyvinylidene fluoride or polyvinylidene difluoride membrane. The transfer was conducted for 90 min at 220 constant mA. The membrane was blocked (overnight at 4 C) using a blocking buffer. Then, it was washed 3 times in tris-buffered saline with 0.1% tweenⓇ 20 detergent for 5 min each. The membrane was incubated with appropriate dilutions of primary antibodies against p-c-Fos (1/1000, Santa Crus Biotechnology, USA) and p-c-Jun (1/1000, Santa Crus Biotechnology, USA) in a blocking buffer at room temperature for 3 hr. It was then washed and incubated with conjugated secondary antibodies in blocking buffer at room temperature for 1 hr. An enhanced chemiluminescence reagent (GE Healthcare, USA) was used to expose the blots to x-ray film (Fuji, Japan). ImageJ software (version 2020) was used to evaluate the blot density (16).

### Ethical considerations

All experiments followed the guidelines on ethical standards for the investigation of experimental pain in animals and were approved by the relevant Ethical Committee of Shahid Bahonar University of Kerman, Kerman, Iran (Code: IR.UK.VETMED.REC.1400.016).

### Statistical analysis

The results were analyzed by Statistical Package for the Social Sciences, SPSS Inc., Chicago, Illinois, USA (version 19). The data were examined using one or two-way ANOVA followed by Tukey's test. All values were reported as the mean ± standard error. A p < 0.05 was considered statistically significant.

## 3. Results

### Formalin test

**Figure 1:**
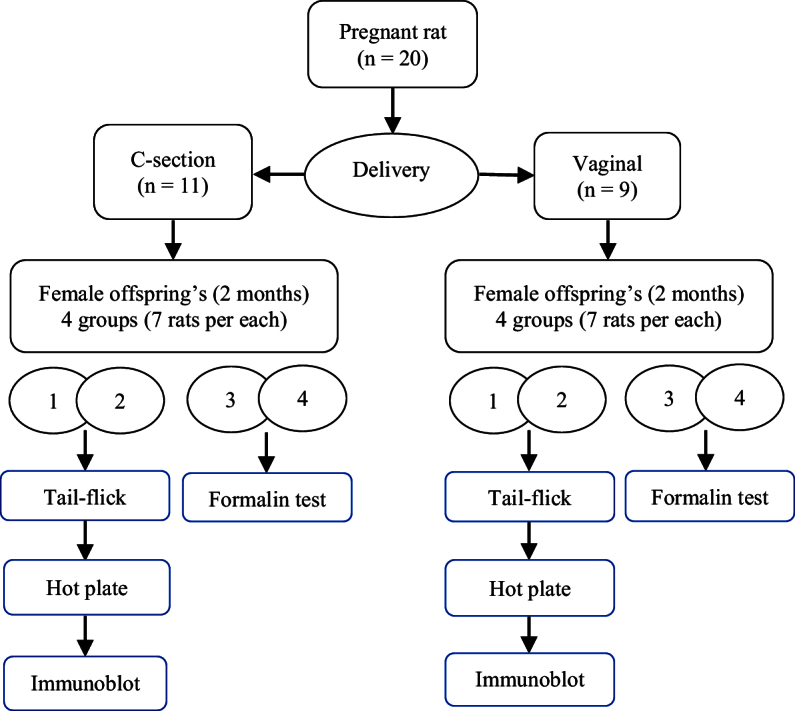
The study flow diagram.

**Figure 2:**
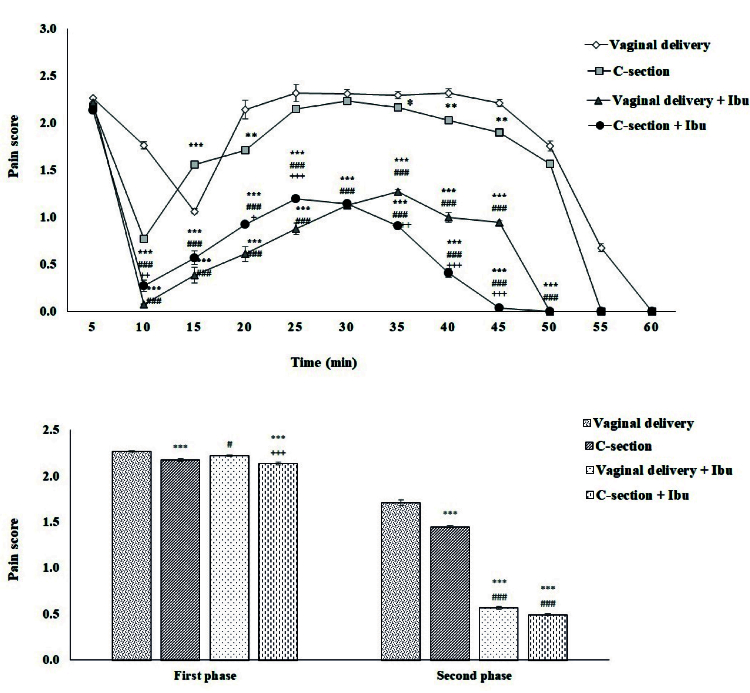
Time course of formalin-induced nociception during 60 min test period (upper graph) and evaluation the mean of nociceptive scores in the first and second phases between groups (lower graph). Values represent Mean ± S.E.M. (n = 7 rats per group). ***P < 0.001, **P < 0.01, *P < 0.05 compared to a vaginal delivery group in the same phase. ${\;}^{\#\#\#}$ P < 0.001, ${\;}^{\#}$ P < 0.05 vs. C-section group in the same phase. ${\;}^{+++}$ P < 0.001 vs. vaginal delivery ${\;}^{+}$ Ibu group. Ibu: Ibuprofen.

The animals in each group were subjected to different experiments as provided in the flow diagram (Figure 1). The time courses of formalin-induced pain in the vaginal delivery and C-section rats during a 60 min test period are shown in figure 2A. In the first phase, formalin-induced pain was significantly decreased in the C-section (2.17 ± 0.013) group compared to vaginal delivery rats (2.26 ± 0.009) (p < 0.001). Also, pre-treatment with intraperitoneal infusion of ibuprofen (10 mg/kg) decreased formalin-induced pain scores in C-section but not vaginal delivered rats (Figure 2B). In the second phase, the C-section group showed lower pain scores (1.45 ± 0.014) than the vaginal delivery group (1.71 ± 0.03) (p < 0.01). Pre-treatment with ibuprofen decreased pain scores in both C-section (0.495 ± 0.011) and vaginal delivery (1.451 ± 0.014) groups (p < 0.001). However, the ibuprofen induced more analgesic effects in the C-section than vaginal delivered rats (Figure 2B).

### Thermal nociceptive tests

**Figure 3:**
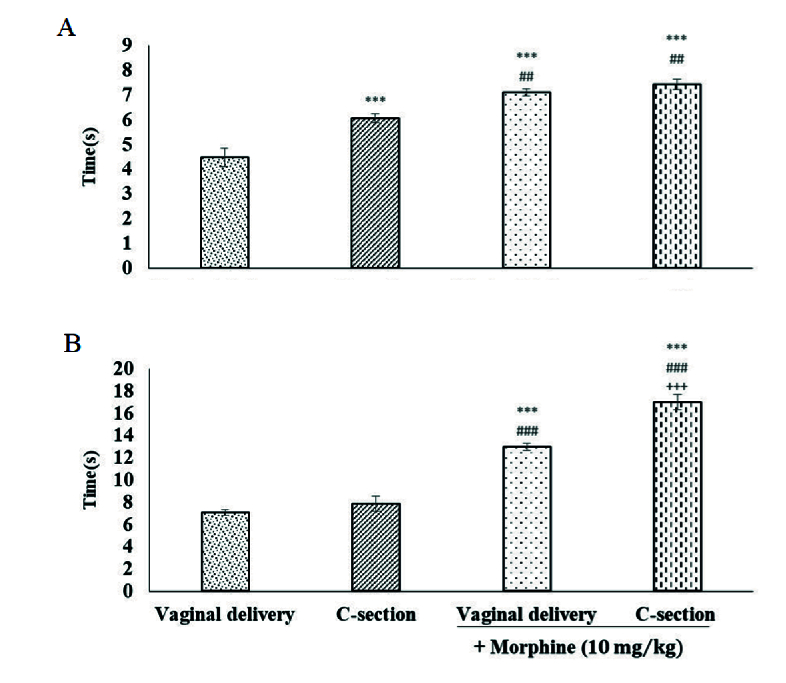
Evaluation of basal nociceptive threshold and morphine induced-analgesia in tail flick A) and hot plate B) tests between vaginal and C-section delivery groups. Values represent Mean ± S.E.M. (n = 7 rats per group). ***P < 0.001 as compared with vaginal delivery, ${\;}^{\#\#}$ P < 0.01, ${\;}^{\#\#\#}$ P < 0.001 vs. C-section + morphine group, ${\;}^{+++}$ P < 0.001 vs vaginal delivery + morphine group.

A significant difference was observed in the tail-flick nociceptive withdrawal threshold between the standard and cesarean delivery groups. As shown in figure 3A, the basal nociceptive threshold increased in the C-section group (5.85 ± 0.63) compared to the vaginal delivery group (4.6 ± 0.42) (p < 0.001). Morphine treatment (10 mg/kg, i.p.) elicited a significant increase, but at the same maximum level, in pain threshold in both experimental groups (Figure 3).

In the hot plate test, the nociceptive threshold did not alter in the C-section group compared to vaginal delivery rats. Morphine pre-treatment caused a significant increase in the baseline nociceptive thresholds in both vaginal (13.6 ± 0.307) (p < 0.05) and C-section (16.66 ± 0.95) (p < 0.001) rats. However, morphine-induced analgesia was more potent in the C-section delivered rats (p < 0.001) (Figure 3B).

### Immunoblot test

**Figure 4:**
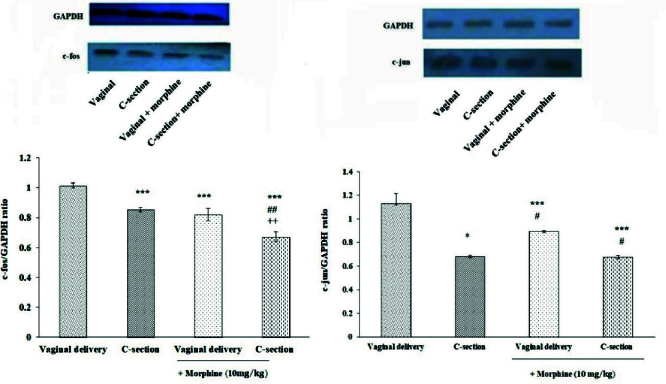
Assessment of the levels of c-Fos and c-Jun proteins in the spinal cord of control and morphine-treated vaginal and C-section rats. Values represent Mean ± S.E.M. ***P < 0.001, *P < 0.05 vs. vaginal delivery, ${\;}^{\#\#}$ P < 0.01, ${\;}^{\#}$ P < 0.05 vs. C-section, ${\;}^{++}$ P < 0.01, ${\;}^{+}$ P < 0.05, vs. vaginal delivery + morphine group.

Immunoblot analysis showed a significant decrease in c-Fos and c-Jun protein values in the lumbar spinal cord of C-section delivered rats compared to vaginal delivery animals (p < 0.001). Besides, c-Fos protein levels were significantly decreased in morphine-treated C-section (0.672 ± 0.032) and vaginal delivery groups (0.821 ± 0.041) (p < 0.001). Morphine-reduced c-Fos protein value was more potent in C-section rats than vaginally delivered group. However, C-section (0.677 ± 0.012) and vaginal delivered (0.895 ± 0.04) rats pretreated with morphine did not show significant differences in the c-Jun protein level (Figure 4).

## 4. Discussion

The present study results indicated that basal sensitivity to thermal and inflammatory stimuli is decreased in the female adult rats born by C-section compared to vaginally delivered rats. Also, morphine-induced thermal analgesia was more noticeable in the C-section group. Moreover, the spinal cord level of c-Fos protein increased in normal and morphine-treated C-section rats compared to the vaginal delivery group. The level of c-Jun decreased in the control group but not in the morphine-treated C-section group compared with vaginal birth rats.

This study showed an association between mode of delivery and changes in pain perception in adult female offspring rats. Some previous studies are available for comparison with the present results. In contrast, capsaicin-evoked pulpal nociception has been shown to decrease in C-section male rats compared to vaginally delivered animals (9). However, a human study demonstrated that spontaneous pain behaviors and vocalization are reduced in infants born vaginally compared to C-section (17). Besides, the recent prospective cohort study revealed no significant differences between vaginal and C-section newborns in acute pain sensation (18).

In this study, the baseline nociceptive threshed and morphine-induced analgesia increased in female C-section rats. The exact mechanism(s) of lower pain sensitivity in C-section rats is not clear. However, differences in stress hormones during childbirth might be considered as one of many possible involving mechanisms. Vaginal delivery as major stress is associated with extensive changes in the number of stress hormones. It has been reported that the stress and pain response of neonates after vaginal birth by vacuum extraction is increased compared to C-section delivery (19). Exposure to endocrine stressors during vaginal childbirth alters the activity of the hypothalamus-pituitary axis in the later life (20). It may increase the risk of pathophysiologic situations such as high blood pressure and metabolic syndromes in the postnatal period.

The level of stress hormones, including adrenocorticotropin and cortisol, in mothers and neonates is significantly higher following spontaneous vaginal delivery compared to C-section birth (21). It reinforces the hypothesis that exposure to different levels of endogenous stressors during vaginal or C-section delivery may be reflected by a long-term effect on hypothalamus-pituitary axis function. It may alter emotional behaviors and stress responsiveness. Cesarean deliveries have been associated with many changes in the physiology and behavior of the offspring (22). Interestingly recent data findings show sex-dependent differences in C-section offspring mice (23).

c-Fos, is a specific marker of neuronal activity that is expressed in nociceptive pathways rapidly after noxious stimuli. It forms a complex with Jun, another oncogene protein, making connections with the activator protein 1 DNA site to induce gene transcription. c-Fos expression after noxious stimulation and tissue injury has been associated with activation of intracellular pathways involved in pain sensitization including PERK/MAPK (24). In this study, to endorse the results of behavioral tests, alteration in the c-Fos and c-Jun proteins were evaluated in the spinal cord of rats. In line with behavioral data, the levels of c-Fos and c-Jun proteins were decreased in control and C-section morphine-treated rats in comparison to vaginally delivered rats. In a related study, c-Fos expression has been increased in the piriform cortex, caudate nucleus, putamen and cerebellar cortex in vaginally delivered compared to C-section born mice (25). These data show mode of delivery interferences on neuronal exhibition as well as nociceptive development.

The circulating concentrations of cytokines like tumor necrosis factors alpha and interleukin 6 in mothers before delivery and infants' in the first 4 days of life have increased in vaginal delivery compared to elective C-sections (26). The human study on maternal and fetal plasma prostaglandin levels showed that the plasma concentration of prostaglandin F is significantly elevated at vaginal delivery compared to C-section (27). The positive correlation between nociceptive and pro-inflammatory responses raised the possibility that C-section rats' low sensitivity to nociceptive impulses could attenuate the inflammatory pathways.

Optimum oxygen supply is essential during parturition. It has been shown that oxygen deprivation during prolonged vaginal delivery increases hypoxia-ischemia-induced brain deficit that could be associated with long-lasting neurological inadequately (28, 29).

It has been indicated that hypoxia, even for a short time*, *may induce oxidative stress damage (30). Studies have shown that oxidative stress plays a vital role in the induction and preserves various pain conditions (31–33). The levels of antioxidant enzymes have been increased in umbilical cord blood of neonates born by elective C-sections compared to those of vaginally born neonates (21). So, mode of delivery-induced changes in pain sensitivity may be somewhat related to alteration in antioxidant enzyme activities. However, further studies are still required to approve the association between delivery mode and the variation in antioxidant enzymes.

## 5. Conclusion

Totally, the present study provides some evidences to support intervention of delivery mode on development of nociceptive behavior and alteration of basal neuronal activities in adults' female rats. However, further research is still required to clarify the intricate underling mechanism(s).

##  Data availability

Data supporting the findings of this study are available upon reasonable request from the corresponding author.

##  Author contributions

All the authors had full access to all of the data in the study and take responsibility for the integrity of the data and the accuracy of the data analysis. Concept and design: Mehdi Abbasnejad, acquisition, analysis, or interpretation of data: Razieh Kooshki, Parastoo Nikkhouy, drafting of the manuscript: Parastoo Nikkhouy, critical revision of the manuscript for important intellectual content: all authors, statistical analysis: Saeed Esmaeili-Mahani, supervision: Razieh Kooshki.

##  Acknowledgments 

The authors would like to thank Shahid Bahonar University, Kerman, Iran for financial support.

##  Conflict of Interest

The authors declare that there is no conflict of interest.

## References

[B1] Negrini R, da Silva Ferreira RD, Guimarães DZ (2021). Value-based care in obstetrics: Comparison between vaginal birth and caesarean section. BMC Pregnancy Childbirth.

[B2] Chen H, Tan D (2019). Cesarean section or natural childbirth? Cesarean birth may damage your health. Front Psychol.

[B3] Barba-Müller E, Craddock S, Carmona S, Hoekzema E (2019). Brain plasticity in pregnancy and the postpartum period: Links to maternal caregiving and mental health. Arch Womens Ment Health.

[B4] Vasung L, Turk EA, Ferradal SL, Sutin J, Stout JN, Ahtam B, et al (2019). Exploring early human brain development with structural and physiological neuroimaging. NeuroImage.

[B5] Fenton BW, Shih E, Zolton J (2015). The neurobiology of pain perception in normal and persistent pain. Pain Manag.

[B6] Dahlquist K, Stuart A, Källén K (2022). Planned cesarean section vs planned vaginal delivery among women without formal medical indication for planned cesarean section: A retrospective cohort study of maternal short‐term complications. Acta Obstet Gynecol Scand.

[B7] Kristensen K, Henriksen L (2016). Cesarean section and disease associated with immune function. J Allergy Clin Immunol.

[B8] Flick RP, Lee K, Hofer RE, Beinborn CW, Hambel EM, Klein MK, et al (2011). Neuraxial labor analgesia for vaginal delivery and its effects on childhood learning disabilities. Anesth Analg.

[B9] Mohamadi-Jorjafki E, Abbasnejad M, Kooshki R, Esmaeili-Mahani S, Raoof M (2020). Mode of delivery alters dental pulp nociception and pain-induced changes in cognitive performance in adults male rats. Can J Physiol Pharmacol.

[B10] Tork Zahrani S, Heshmat F, Abbasinia H, Delshad H, Shakeri N, Valiani M (2021). The relationship between delivery mode and cord blood betaendorphins values in the newborns of nulliparous women. Arc Hygiene Sci.

[B11] Li Sh, Ye F, Farber JP, Linderoth B, Zhang T, Gu JW, et al (2019). Dependence of c-fos expression on amplitude of high-frequency spinal cord stimulation in a rodent model. Neuromodulation.

[B12] Li Y-L, Chang X-R, Ma J-T, Zhao X, Yin L-T, Yan L-J, et al (2022). Activation of peripheral group III metabotropic glutamate receptors suppressed formalin‐induced nociception. Clin Exp Pharmacol Physiol.

[B13] Zhou F, Zhang W, Zhou J, Li M, Zhong F, Zhang Y, et al (2017). Involvement of endoplasmic reticulum stress in formalin-induced pain is attenuated by 4-phenylbutyric acid. J Pain Res.

[B14] Askari-Zahabi Kh, Abbasnejad M, Kooshki R, Raoof M, Esmaeili-Mahani S, Pourrahimi AM, et al (2022). The role of basolateral amygdala orexin 1 receptors on the modulation of pain and psychosocial deficits in nitroglycerin-induced migraine model in adult male rats. Korean J Pain.

[B15] Deuis J, Dvorakova LS, Vetter I (2017). Methods used to evaluate pain behaviors in rodents. Front Mol Neurosci.

[B16] Sule R, Rivera G, Gomes AV (2023). Western blotting (immunoblotting): History, theory, uses, protocol and problems. Biotechniques.

[B17] Bergqvist LL, Katz-Salamon M, Hertegård S, Anand K, Lagercrantz H (2009). Mode of delivery modulates physiological and behavioral responses to neonatal pain. J Perinatol.

[B18] Ferreira EAL, Nassif DS, Silva MJD, Barbosa SMdM, Módolo NSP, Barros GAM (2020). [Evaluation of pain in vaginal and caesarean section birth newborns before and after intramuscular injection]. Braz J Pain.

[B19] Schuller Ch, Känel N, Müller O, Kind AB, Tinner EM, Hösli I, et al (2012). Stress and pain response of neonates after spontaneous birth and vacuum-assisted and cesarean delivery. Am J Obstet Gynecol.

[B20] Glover V, O’connor T, O’Donnell K (2010). Prenatal stress and the programming of the HPA axis. Neurosci Biobehav Rev.

[B21] Kaviany Nejad R, Goodarzi MT, Shfiee Gh, Pezeshki N, Sohrabi M (2016). Comparison of oxidative stress markers and serum cortisol between normal labor and selective cesarean section born neonates. J Clin Diagn Res.

[B22] Cabré S, Ratsika A, Rea K, Stanton C, Cryan JF (2022). Animal models for assessing impact of C-section delivery on biological systems. Neurosci Biobehav Rev.

[B23] Chiesa M, Ferrari DC, Ben-Ari Y (2020). Alteration in the time and/or mode of delivery differentially modulates early development in mice. Mol Brain.

[B24] Gao Y-J, Ji R-R

[B25] Ikeda K, Onimaru H, Matsuura T, Kawakami K (2019). Different impacts on brain function depending on the mode of delivery. Brain Res.

[B26] Malamitsi-Puchner A, Protonotariou E, Boutsikou T, Makrakis E, Sarandakou A, Creatsas G (2005). The influence of the mode of delivery on circulating cytokine concentrations in the perinatal period. Early Hum Dev.

[B27] Palm M, Axelsson O, Wernroth L, Larsson A, Basu S (2013). Involvement of inflammation in normal pregnancy. Acta Obstet Gynecol Scand.

[B28] Diaz J, Abiola S, Kim N, Avaritt O, Flock D, Yu J, et al (2017). Therapeutic hypothermia provides variable protection against behavioral deficits after neonatal hypoxia-ischemia: A potential role for brain-derived neurotrophic factor. Dev Neurosci.

[B29] Ayres-de-Campos D, Ayres-de-Campos D Obstetric emergencies: A practical guide.

[B30] Panfoli I, Candiano G, Malova M, De Angelis L, Cardiello V, Buonocore G, et al (2018). Oxidative stress as a primary risk factor for brain damage in preterm newborns. Front Pediatr.

[B31] Ilari S, Giancotti LA, Lauro F, Gliozzi M, Malafoglia V, Palma E, et al (2020). Natural antioxidant control of neuropathic pain-exploring the role of mitochondrial SIRT3 pathway. Antioxidants.

[B32] Sharma A, Kumar A (2021). Role of antioxidant therapy for pain relief in chronic pancreatitis: Finding the signal in the noise. JGH Open.

[B33] Kaushik AS, Strath LJ, Sorge RE (2020). Dietary interventions for treatment of chronic pain: Oxidative stress and inflammation. Pain Ther.

